
JOURNAL INFORMATION
==============================
NLM Title Abbreviation: ScientificWorldJournal
Journal ID: ScientificWorldJournal
NLM Journal ID: 101131163
Journal ID: TSWJ

The Scientific World Journal

ISSN: 2356-6140
EISSN: 1537-744X
Publisher: Wiley

ARTICLE INFORMATION
==============================
PMCID: PMC6084099
PMID: 30147475
DOI: 10.1100/tsw.2001.162
Article ID: 471247
Article version: 1
Subjects: Short Report

A Novel P53-Related Activity in a Colon Adenocarcinoma Cell Line With Mutant P53

Gartel Andrei L. *
Goufman Eugene
Hurth Charlotte
Tyner Angela L.
Department of Molecular Genetics, University of Illinois at Chicago, 900 S. Ashland Ave., Chicago, IL 60607, USA
*Andrei L. Gartel: agartel@uic.edu
Publication date: 2001
Electronic publication date: 2001 Oct 23
Volume: 1
First page: 36
Last page: 36
Copyright: Copyright © 2001 Andrei L. Gartel et al.
Copyright year: 2001
License: This is an open access article distributed under the Creative Commons Attribution License, which permits unrestricted use, distribution, and reproduction in any medium, provided the original work is properly cited.
License URL: https://creativecommons.org/licenses/by/3.0/

Funding: National Institutes of Health DK48836

==============================
Miami *Nature Biotechnology* Short Reports
*TheScientificWorld* (2001) 1(S3), 36SR
ISSN 1532-2246; DOI 10.1100/tsw.2001.162

### A NOVEL P53-RELATED ACTIVITY IN A COLON ADENOCARCINOMA CELL LINE WITH MUTANT P53

Andrei L. Gartel*, Eugene Goufman, Charlotte Hurth, and Angela L. Tyner

Department of Molecular Genetics, University of Illinois at Chicago, 900 S. Ashland Ave., Chicago, IL 60607
* agartel@uic.edu

**INTRODUCTION.** Caco-2 cells were derived from a human colon adenocarcinoma and they spontaneously differentiate in the absence of inducers after reaching confluence. These cells lack functional p53. We demonstrate here that Caco-2 cells have a p53-related DNA-binding and transactivator activity that is not p53 or p73.

**METHOD.** CAT reporters with different p53 binding sites including E2-70- Shay CAT, WAF1-TK-CAT and mdm2-TK-CAT were received from: M. Tarunina (M. Curie Res. Institute) and R. Kondratov (Univ. of Illinois, Chicago) respectively. p53 mutants R175H and R248W in pCMV-neo-Bam vector were received from C.J. Di Como (Columbia University). Caco-2 cells were obtained and transfected as described earlier (1). Electrophoretic Mobility Shift Assays (EMSAs) were performed as described in (1).

**RESULTS.** We performed EMSAs with a double stranded oligonucleotide representing a proximal p53-binding site from the p21 promoter as a probe and with nuclear extracts from undifferentiated or differentiated Caco-2 cells. We found that this probe binds specifically to protein complexes [1] and nonspecifically to protein complexes [2] from undifferentiated and differentiated Caco-2 cells. The mobility of the specific DNA-protein complexes was different than the mobility of similar DNA-protein complexes from COS-1 cells, which contain wild type p53. A supershift assay demonstrated that antisera specific for p53 could supershift the complex formed with extracts from COS-1 cells, but not the complex from Caco-2 cells. Antibodies specific for p73 could not supershift complexes from Caco-2 or COS-1 cells. Caco-2 cells appear to have a p53-related DNA-binding activity that is neither p53 nor p73.

To further study this activity we introduced plasmids with different p53-responsive elements upstream of the CAT reporter into Caco-2 cells and compared their activity with activity of the same reporters lacking p53 binding sites. We used three different p53-responsive elements: Shay, GGACATGCCCG GGCATGTCC (E2-70-Shay-CAT); the distal p53 binding site from the human p21 promoter, GAACATGTCCCAACATGTTG (WAF1-TK-CAT); and the p53 binding site from the mdm2 promoter that is proximal to TATA box, AGCTAAGTCCTGACATGTCT (mdm2-TK-CAT). All three p53 cis-acting elements are active in Caco-2 cells. An oncogenic dominant negative p53 mutant R175H, with a mutation within the DNA-binding domain (2) inhibits transactivation of p53 responsive elements by both p53 (2,3) and its structural and functional homolog p73 (3). An oncogenic dominant negative mutant R248W also can inhibit transactivation of p53 responsive elements by both p53 (2) and p73 (3). Cotransfection of these dominant negatives p53 mutants together with p53-responsive reporters into Caco-2 cells did not affect transactivation of these reporters. These data support the notion that the p53-related transactivator from Caco-2 cells is not p53 or p73 protein.

**DISCUSSION.** We have identified a novel p53-related DNA-binding activity in Caco-2 cells and we found that dominant negative p53 mutants R175H and R248W (2) do not interfere with the p53-related transcriptional activity in Caco-2 cells, suggesting that p53 and p73 are not responsible for transactivation of p53-binding sites. Several additional p53-related proteins have been identified including p44 (4), p63 (5), p51 (6) and some others. One of these proteins or an unidentified protein may be responsible for p53-related transcriptional and DNA-binding activity in Caco-2 cells.

**ACKNOWLEDGEMENTS.** This work was supported by awards #97-01 and #98-28 from the ACS, Illinois Division (A.L.G), NIH Grant DK48836 (A.L.T.) and by NCI Oncology Research Faculty Development Program (E.G.).

**REFERENCES.**

1. Gartel, A.L., Goufman, E., Najmabadi, F., and Tyner, A.L. (2000) Oncogene 19, 5182-5188
2. Kern, S.E., Pietenpol, J.A., Thiagalingam, S., Seymour, A., Kinzler, K.W., and Vogelstein, B. (1992) Science 256, 827-830
3. Di Como, C.J., Gaiddon, C., and Prives, C. (1999) Mol. Cell. Biol. 19, 1438-1449
4. Zeng, X., Levine, A.J., and Lu, H. (1998) Proc. Natl. Acad. Sci. U S A 95, 6681-6686
5. Yang, A., Kaghad, M., Wang, Y., Gillett, E., Fleming, M.D., Dotsch, V., Andrews, N.C., Caput, D., and McKeon, F. (1998) Mol. Cell 2, 305-316
6. Osada, M., Ohba, M., et al. (1998) Nat. Med. 4(7), 839-843
